# Longitudinal assessment of SNPs rs72552763 and rs622342 in *SLC22A1* over HbA1c control among Mexican-Mestizo diabetic type 2 patients

**DOI:** 10.3389/fphar.2024.1433519

**Published:** 2024-09-30

**Authors:** Adiel Ortega-Ayala, Fernando De Andrés, Adrián Llerena, Carlos Miguel Bartolo-Montiel, Gustavo Acosta-Altamirano, Juan Arcadio Molina-Guarneros

**Affiliations:** ^1^ Department of Pharmacology, Faculty of Medicine, National Autonomous University of Mexico, Mexico City, Mexico; ^2^ Department of Analytical Chemistry and Food Technology, Faculty of Pharmacy, University of Castilla-La Mancha, Albacete, Spain; ^3^ Directorate of University Institute for Bio-Sanitary Research of Extremadura, Badajoz, Spain; ^4^ Directorate of Planification, Teaching, and Research, High-Speciality Regional Hospital of Ixtapaluca, Ixtapaluca, Mexico; ^5^ Dirección de Investigación, Hospital General de México, Dr. Eduardo Liceaga, Mexico City, Mexico

**Keywords:** rs72552763, rs622342, HbA1c control, metformin, diabetes T2

## Abstract

**Background:** In Mexico, 75% of diabetes mellitus type 2 (DMT2) patients are not in glycaemic control criteria (HbA1c<7%); this entails a significantly variable drug response. Amongst the factors influencing such variability, are genetics, more specifically, single nucleotide polymorphisms (SNPs). Three genes implied in metformin pharmacokinetics are *SLC22A1*, *SLC22A2*, and *SLC22A3*, which are polymorphic. While there have been cross-sectional studies on their SNPs impact over drug response, a longitudinal study would contribute valuable information on their effect over time.

**Methods:** SNPs of *SLC22A1* (rs72552763, rs622342, rs12208357, rs2282143, rs594709, rs628031, and rs683369), *SLC22A2* (rs316019), and *SLC22A3* (rs2076828), were determined through PCR-TR. The clinical records of 69 patients undergoing metformin monotherapy were retrospectively assessed. Metformin is the first line treatment against DMT2. A level of HbA1c <7% (time 0) was considered as an inescapable inclusion criterion. The study’s cases were those patients who reported HbA1c ≥ 7% (time1) after time 0 (t0). Kaplan-Meier curves including a Log-Rank test and a Cox multivariate analysis of proportional risks were performed.

**Aim:** Determining clinical, biochemical, and genetic variables which may affect non-control (HbA1c ≥ 7%) survival time spans amongst DMT2 Mexican-Mestizo patients undergoing metformin monotherapy at *Hospital Regional de Alta Especialidad de Ixtapaluca* (HRAEI) between October 2013 and December 2023.

**Results:** All 69 patients were monitored over a median period of 642 days (273-1,134). A comparison between time 0 and time 1 (t1) revealed differences in weight (*p* = 0.036), metformin dose mg/kg/day (*p* = 0.003), plasmatic glucose mg/dL (*p* = 0.048), and HbA1c (*p* < 0.001). The median non-control survival rate was different across the 3 genotypes of rs62552763 in *SLC22A1* (*p* = 0.0034) and the dominant genotypic model GAT/GAT vs. GAT/del + del/del (*p* = 0.009). There were differences between rs622342 genotypes as well (*p* = 0.041). In GAT/GAT the Cox model found HR = 0.407 (IC95%: 0.202–0.818, *p* = 0.011) in the univariate analysis and HR = 0.418 (IC95%: 0.204–0.856, *p* = 0.034) in the multivariate analysis, adjusted by initial metformin dose (mg/kg/day), initial weight (kg), and final metformin dose (mg/kg/day). Genotype A/A of rs622342 in *SLC22A1*, reported HR = 0.392 (IC95%: 0.169–0.910, *p* = 0.029) in the multivariate analysis as well.

**Conclusion:** Among DMT2 Mexican-Mestizo patients undergoing metformin monotherapy the minor allele del in rs72552763 and the minor allele C in rs622342 reported a significantly shorter survival median respect to the wild type variant. Patients carrying del in rs72552763 or C in rs622342, both in *SLC22A1*, will reach non-control in less time with respect to other patients. Therefore these genotypes may constitute a therapeutic response biomarker for this population.

## 1 Introduction

In its political constitution, Mexico defines itself as a multicultural nation based originally on its indigenous peoples, described as descendants of those inhabiting the country before colonisation and preserving their own social, economic, cultural, and political institutions, or some of them (México, 2013). Considering the widely heterogeneous ethnic background of Mexican populations (which comprise more than 65 groups), the study of their biological variability is evident (Jimenez-Sanchez, 2003). A recent article on Mexican Biobank advances, population, and medical genomics of diverse ancestries conducted genome-wide association studies (GWAS) for 22 complex traits, finding that several of them were better predicted using the Mexican Biobank GWAS as compared to the UK Biobank GWAS, and also identifying genetic and environmental factors associated with trait variation, such as the genome’s length in runs of homozygosity as a predictor for body mass index, triglycerides, glucose, and height (Sohail et al., 2023). While this particular GWAS survey provides insights into the genetic histories of individuals in Mexico and dissects their complex trait architectures, two crucial conditions for making precision and preventive medicine initiatives accessible worldwide, it is insufficient to further our knowledge of complex diseases such as DMT2 and its behaviour across every Mexican ethnic group and geographic region. This raises the importance of observational studies like the present work, which focuses on a small cohort of patients affected by this chronic disease.

On 14 November 2016, the Mexican Secretary of Health declared diabetes and obesity a national epidemiological emergency, a status that prevails today (CENAPRESE, 2018). Mexico holds the sixth largest global DMT2 population, amounting to 8.7 million people. It is estimated that by 2035, this will rise to the fifth place with a total of 15.7 million (20–79 years of age) (Guariguata et al., 2014). According to the International Diabetes Federation (IDF), the adjusted prevalence was 13.1% between the 20–79-year-old group in 2017 (International Diabetes Federation, 2021).

In Mexico, the National Institute for Public Health has carried out the National Health and Nutrition Survey (ENSANUT) for more than 25 years. According to the latest data of ENSANUT 2021 COVID-19, the prevalence of diabetes was 11.1% (confidence interval 95% = 9.5–12.8) in a country whose population was 128.9 million in 2020 (Shamah-Levy et al., 2022).

Metformin is classified as a biguanide and it is considered an essential drug by the WHO. It is the first line therapy against DMT2 because of its efficacy, safety, and low cost. It has a beneficial effect on HbA1c and weight reduction, and it can reduce cardiovascular and death risk (Turner, 1998). Metformin’s primary effect is the hepatic inhibition of gluconeogenesis; yet, its mechanism is still debated (Lamoia and Shulman, 2021). Metformin is a positively charged hydrophilic molecule that is mainly transported by organic cations 1–3 (OCT1-3), equilibrant nucleoside transporter 4 (ENT4), and multidrug and toxin extrusions (MATEs) 1 and 2k (Lamoia and Shulman, 2021; Staiger et al., 2015).

Transporters involved in metformin pharmacokinetics and pharmacodynamics belong to the solute carrier (SLC) family. This superfamily encompasses OCTs. OCT1 is a 554 amino acid protein with a molecular weight of 61,154 Da; it is coded by gene *SLC22A1*, located in the long arm of chromosome 6 in region 25.3 (6q25.3), possessing a total of 12 exons (Weizzman Institute of Science, 2022). Like numerous other members of the *SLC22* family, it has 12 helixes or transmembrane domains (TMDs), including a large extracellular loop between domains 6 and 7 (Koepsell et al., 2007). *SLC22A1* is a highly polymorphic gene and its polymorphisms may induce altered OCT1 function, which affects metformin pharmacokinetics and response (Staiger et al., 2015; Lai, 2013). OCT2 is a 555 amino acid transporter coded by *SLC22A2* (Zazuli et al., 2020). It is in charge of renal metformin uptake through renal tubular cells (Staiger et al., 2015; Zazuli et al., 2020) and it partakes up to 80% of its renal clearance (Emami Riedmaier et al., 2013). Evidence shows that some *SLC22A1* and *SLC22A3* polymorphisms affect metformin pharmacokinetics; association analysis revealed that rs316019 in *SLC22A2* is significantly associated with metformin drug response across co-dominant models (Phani et al., 2018). OCT3 is a 556 amino acid transporter coded by gen *SLC22A3* and it is expressed over a variety of tissues including the liver, skeletal muscle, colon, and heart (Zazuli et al., 2020). It is moderately expressed in renal tubular cells and it is less important than OCT2 in terms of renal cation elimination, although it plays an important role in these compounds’ biliary excretion (Zazuli et al., 2020). Taheri et al. (2022) reported the influence of *SLC22A3* rs543159 and rs1317652 genetic variants on metformin therapeutic efficacy in newly diagnosed patients with type 2 diabetes mellitus. Pharmacokinetic parameters of metformin were found to be affected by age, sex, ethnicity, and several polymorphisms in *ABCG2*, *SLC22A1*, *SLC22A3*, *SLC22A4*, and *SLC47A2* (Saiz-Rodríguez et al., 2023). It is thereby important to further persist in studying the pharmacogenetics of DMT2 patients, expanding our studies towards transporters involved in metformin pharmacokinetics and pharmacodynamics belonging to the solute carrier *(SLC)* family. This superfamily encompasses OCT1, OCT2, and OCT3 polymorphisms upon which new and impactful evidence has arisen, presenting an opportunity to reduce futile biomedical research. The effect of these polymorphisms on metformin therapeutic response among DMT2 patients fosters novel approaches such as the present research that reports clinical, biochemical, and genetic variables which may affect non-control (HbA1c ≥ 7%) survival time spans amongst DMT2 Mexican-Mestizo patients undergoing metformin monotherapy.

## 2 Materials and methods

### 2.1 Genotyping procedure

Genotyping was performed as previously described (Ortega-Ayala et al., 2022). A 10 mL peripheral blood sample was collected from all participants in EDTA tubes, and genomic DNA was extracted from 200 μL venous peripheral blood using UltraClean^®^ BloodSpin^®^ DNA isolation reagents (Mo Bio Laboratories; Qiagen, Inc.), evaluated for integrity and concentration via 1% agarose electrophoresis and spectrophotometry using NanoDrop™ 2000/2000c (Thermo Scientific, Inc.), respectively. For *SLC22A1*, different allelic variants were analysed through real time PCR technology using fluorescence based TaqMan^®^ assays on a Fast 7300 Real Time PCR System (both Applied Biosystems; Thermo Fisher Scientific, Inc.). Reactions were performed in a final reaction volume of 10 μL with 30 ng of genomic DNA template, 1X TaqMan^®^ Universal PCR Master mix, 1X each probe assessed (*SLC22A1*: rs12208357, C__30634096_10; *SLC22A1*: rs2282143, C__15877554_40; *SLC22A1*: rs594709, C___1898206_20; *SLC22A1*: rs622342, C____928527_30; *SLC22A1*: rs628031, C___8709275_60; *SLC22A1*: rs683369, C____928536_30; *SLC22A1*: rs72552763, C__34211613_10, *SLC22A2*: rs316019, C__3111809_20; *SLC22A3*: rs2076828, C__2763995_1) and water. Thermocycling conditions and genotyping allelic discrimination using ABI PRISM 700 Sequence Detection System v1.0 software (Applied Biosystems; Thermo Fisher Scientific, Inc.) were as previously described (Ortega-Ayala et al., 2022). SNP allelic and genotypic frequencies of OCT1, OCT2, and OCT3 were performed by direct counting.

### 2.2 Study design

This is an observational, longitudinal, clinical, analytical, and retrospective study, carried out at the High Specialty Regional Hospital of Ixtapaluca (HRAEI), Ixtapaluca, Mexico. We surveyed a cohort of 103 DMT2 Mexican-Mestizo patients undergoing metformin monotherapy or a combined metformin + glibenclamide treatment (Ortega-Ayala et al., 2022). All patients did define themselves as Mestizo and all of them were unrelated. We reviewed a total of 103 electronic clinical records from the aforementioned sample, dating back to October 2013. The screening took place between November-December 2023. Out of the original sample, 69 patients met inclusion criteria.

#### 2.2.1 Inclusion and exclusion criteria

Inclusion criteria were i) patients undergoing metformin monotherapy, ii) patients in evident glycaemic control (HbA1c <7%), iii) individuals treated as outpatients of internal medicine, endocrinology, geriatrics, or cardiology at HRAEI, iv) patients with a DMT2 diagnosis (ADA 2024) (Association, 2024). Exclusion criteria were i) any treatment different from metformin monotherapy, ii) no clinical record on HbA1c, iii) hepatic or pancreatic disease and kidney failure.

#### 2.2.2 Basal and monitoring characteristics

A survival analysis requires case criteria absence by every participant, therefore all patients had to report HbA1c<7%; in other words, all 69 patients had to be in glycaemic control. Once HbA1c control had been established, the patient’s record was reviewed, focusing on laboratory studies reporting HbA1c ≥ 7%, which turned the patient into a case. We then proceeded to mark the date for the survival analysis. Patients who reported no evidence of non-control were considered censored cases. We marked the last available control date in order to estimate the control period. A survival analysis requires cases and censorship, because both kinds of patients contribute analysis periods, thereby the Kaplan-Meier curves’ and Cox models’ vigour with respect to time. The survival median and 95% confidence intervals were obtained. For the purposes of the longitudinal analysis, cases were defined as those patients who reported evidence of HbA1c ≥ 7% since the monitoring’s beginning (t0) throughout the HRAEI electronic clinical record, whereas those patients reporting no evidence of non-control were considered as censored cases. The last available HbA1c measurement (t1) accounted for the survival analysis.

#### 2.2.3 Data collection

Clinical and biochemical characteristics were gathered directly from the clinical files using Microsoft Excel 2019. A second participant randomly filled out the data of 10 patients to control and verify clinical data. A second database contained the information on genetic polymorphisms; access to this second database was not available before completing the aforementioned clinical and biochemical data of the selected patients.

### 2.3 Statistical analysis

#### 2.3.1 Descriptive analysis

We used R language version 4.2.0 (https://www.R-project.org/) to perform our statistical analyses and to develop the corresponding graphics. Descriptive statistics were accomplished using frequency and percentages for qualitative variables, whilst normalcy tests were carried out for the quantitative variables. Normal distribution variables are shown with median and standard deviation whilst free distribution variables are shown with median and interquartile ranges at 25 and 75 percentiles.

#### 2.3.2 Inferential analysis

The statistical inference of basal (t0) and final (t1) characteristics was performed using the Wilcoxon signed-rank test for quantitative variables and the McNemar test for dichotomous qualitative variables. For the quantitative variable analysis we used the Shapiro-Wilk normality test to determine data distribution by each genotype. For the quantitative variable inference across 3 groups (SNPs rs72552763 and rs622342) the Kruskal–Wallis test was conducted and followed *post hoc* by Mann-Whitney’s U test. For the inference between t0 and t1, paired Wilcoxon tests were performed, where *p* < 0.05 was statistically significant.

#### 2.3.3 Allelic and genotypic frequency analysis

Genotypes are displayed by frequency and percentage, as well as expected frequencies and allelic frequencies. Hardy-Weinberg equilibrium was obtained through Pearson’s Chi-square test, where *p* > 0.05 was the equilibrium value. We used Hardy Weinberg v.1.7.8 package (https://www.R-project.org/) and Pearson’s Chi square test was calculated with continuity correction and 10,000 permutations (Graffelman and Weir, 2016). Through Pearson’s Chi square test with continuity correction and Monte Carlo simulation at 2000 replicates we compared allelic and genotypic frequencies of rs72552763 and rs622342 against other world populations using the 1000 Genomes Project Phase 3, gnomAD exomes r2.1.1, NCBI ALFA found in Ensemble (Harrison et al., 2024).

#### 2.3.4 Survival analysis

Kaplan-Meier curves were obtained for genotypic variants of *SLC22A1* (rs72552763 and rs622342), *SLC22A2* (rs316019), and *SLC22A3* (rs2076828). After grouping rs72552763 and rs622342 of *SLC22A1*, 4 Kaplan-Meier curves were performed to analyse survival through Log-Rank’s test. Two were carried out on 3 independent groups in accordance with patients’ genotypes, and another two were carried out by grouping the dominant genotypic models of rs72552763 (GAT/GAT vs. GAT/del + del/del) and rs622342 (A/A vs. A/C + C/C). A total of 10 simple models were performed considering the following variables: rs72552763 (GAT/GAT as the reference genotype), initial metformin dose (mg/kg/day), initial body weight (Kg), and final metformin dose (mg/kg/day). The models were conducted across 3 data sets: i) the whole sample (n = 69), ii) GAT/GAT carriers (n = 31), iii) carriers of del (minor allele) in rs72552763 of *SLC22A1* (n = 38); additionally, 3 multiple models were carried out across each group (i, ii, iii). Correspondingly, rs622342 was analysed through 10 simple models and a 3 data set grouping: i) the whole sample (n = 69), ii) A/A carriers (n = 25), iii) carriers of C (minor allele) in rs622342 of *SLC22A1* (n = 44). Multiple models were conducted across each group (i, ii, iii).

## 3 Results

Basal clinical characteristics. Statistic data of the sample’s basal line (n = 69) are summarised in Table 1. There were 25 men (36.23%) and 44 women (63.76%). HbA1c levels were 6.20 (6.00–6.90), where 100% of the patients were in glycaemic control (HbA1c < 7%).

**TABLE 1:** Sample characteristics (time 0).

Characteristic	n = 69
Age, years	54.66 ± 10.04
Sex *Male* *Female*	25 (36.23%) 44 (63.76)
Height, m	1.57 ± 0.08
Weight, kg	76.25 (66.37–83.55)
BMI, kg/m^2^	30.47 (27.14–33.05)
Systolic BP, mmHg	120.00 (110.00–134.75)
Diastolic BP, mmHg	74.00 (70.00–80.00)
Metformin dose, mg/kg/day	18.76 (11.48–23.34)
Fasting glucose, mg/dL	130.00 (111.00–152.00)
HbA1c, %	6.20 (6.00–6.90)
Control HbA1c, (<7%)	69 (100%)
BUN, mg/dL	15.60 (11.95–18.15)
Cr, mg/dL	0.70 (0.60–0.90)
Uric acid, mg/dL	5.70 (4.70–6.35)
Total cholesterol, mg/dL	168.37 ± 48.70
Triglyceride, mg/dL	170.50 (123.25–224.75)
GFR, mL/min/1.73	97.92 (68.73–103.66)
Monitoring period (days)	642 (273–1,134)

Variables with normal distribution are presented as mean and ±standard deviation; variables with non-normal distribution are presented as median and interquartile range 25%–75%. BMI, body mass index; BP, blood pressure; HbA1c, glycated hemoglobin; BUN, blood urea nitrogen; Cr, creatinine; GFR, glomerular filtration rate.

### 3.1 Inference analysis t0 vs. t1

Descriptive statistics up to t1 and Δt1-t0 values are summarised in Table 2. There were statistically significant changes in the clinical traits between t0 and t1 regarding weight (*p* = 0.036), metformin dose (mg/kg/day) (*p* = 0.003), fasting glucose mg/dL (*p* = 0.048), HbA1c levels (*p* < 0.001), and uncontrolled patients (*p* < 0.001).

**TABLE 2:** Sample characteristics at time 1 (t1).

Characteristic	n = 69	Δ_t1-t0_	*p-*value
Weight, kg	74.70 (64.70–82.57)	−0.80 (−6.30, 1.82)	0.036*
BMI, kg/m^2^	30.29 (26.85–32.00)	−0.31 (−2.62, 0.76)	0.063
Systolic BP, mmHg	125.00 (113.00–139.00)	3.00 (−12, 17)	0.347
Diastolic BP, mmHg	75.00 (70.00–80.00)	3.00 (−12, 17)	0.442
Metformin dose, mg/kg/day	22.81 (13.15–30.90)	1.18 (−0.36, 11.44)	0.003*
Fasting glucose, mg/dL	123.00 (102.00–140.00)	−11.00 (−35.00, 12.00)	0.048*
HbA1c, %	7.00 (6.10–7.60)	0.50 (0.00, 1.30)	<0.001*
Control HbA1c, (<7%)	32 (46.37%)	−37 (−53.62%)	<0.001*
BUN, mg/dL	15.80 (11.6–20.30)	0.00 (0.00, 0.44)	0.944
Cr, mg/dL	0.70 (0.60–0.90)	0.0 (−0.1, 0.1)	0.816
Uric acid, mg/dL	5.80 (4.80–6.50)	0.00 (−0.20, 0.42)	0.288
Total cholesterol, mg/dL	172.50 (139.25–191.50)	0.00 (−22.00, 13.50)	0.381
Triglyceride, mg/dL	189.00 (129.25–227.00)	1.50 (−28.00, 46.75)	0.629
GFR, mL/min/1.73	95.95 (66.59–105.71)	0.00 (−5.84, 5.39)	0.844

Median and interquartile ranges (p25-p75) are shown. Δ _t1-t0,_ Difference between the measurement at time 1 and time 0; *Statistical significance (*p* < 0.05). BMI, body mass index; BP, blood pressure; HbA1c, glycated hemoglobin; BUN, blood urea nitrogen; Cr, creatinine; GFR, glomerular filtration rate.

#### 3.1.1 Allelic and genotypic frequencies

The Hardy-Weinberg equilibrium analysis revealed allelic frequencies GAT = 91 (65.94%) and del = 47 (34.05%) by rs72552763 (*p* = 0.593); also, A = 79 (57.25%) and C = 59 (42.75%) by rs622342 (*p* = 0.240). Both SNPs were in Hardy-Weinberg equilibrium (Table 3). We have added a table displaying SNPs of *SLC22A1* (rs12208357, rs2282143, rs594709, rs628031, and rs6833369), rs316019 of *SLC22A2,* and rs2076828 of *SLC22A3* as Supplementary Material S1. Minor allele frequency (MAF) < 5% SNPs were not accounted for in the analysis.

**TABLE 3:** Genotypic and allelic frequencies and HWE of rs72552763 and rs622342 in *SLC22A1*.

SNP	Genotype	Genotypic frequency n (%)	Expected frequency n (%)	Allele frequency n (%)	HWE *χ* ^*2*^ *p-*value	HWE *χ* ^*2*^ ^ǁ^ *p-*value	HWE *exact test* ^ǂ^ *p-*value
rs72552763	GAT/GAT	31 (44.92%)	30.00 (43.48%)	GAT: 91 (65.94%) del: 47 (34.05%)	0.285 0.593	0.110 0.786	0.403 0.590
	GAT/del	29 (42.02%)	30.99 (44.91%)				
	del/del	9 (13.04%)	8.00 (11.59%)				
rs622342	A/A	25 (36.23%)	22.61 (32.77%)	A: 79 (57.25%) C: 59 (42.75%)	1.379 0.240	0.981 0.317	0.770 0.234
	A/C	29 (42.02%)	33.77 (48.94%)				
	C/C	15 (21.73%)	12.61 (18.27%)				

SLC, solute carrier; SNP, single nucleotide polymorphism; HWE, Hardy-Weinberg Equilibrium, χ^2^, Xi statistic. ^ǁ^Chi-square statistic with continuity correction with 10,000 permutations, ^ǂ^statistic exact test with 10,000 permutations.

#### 3.1.2 Allelic ancestry comparison with different world populations

The comparison of allelic frequencies of SNP rs622342 across world populations reported in the 1,000 Genome Project Phase 3, revealed several significant statistical differences: AFR (African; *p* < 0.001), ACB (African Caribbean in Barbados; *p* < 0.001), ASW (African Ancestry in Southwest US; *p* = 0.001), ESN (Esan in Nigeria; *p* < 0.001), GWD (Gambian in Western Division, The Gambia; *p* < 0.001), LWK (Luhya in Webuye, Kenya; *p* = 0.001), MSL (Mende in Sierra Leone; *p* < 0.001), YRI (Yoruba in Ibadan, Nigeria; *p* < 0.001), EAS (East Asian; *p* < 0.001), CDX (Chinese Dai in Xishuangbanna, China; *p* < 0.001), CHB (Han Chinese in Beijing, China; *p* < 0.001), CHS (Southern Han Chinese, China; *p* < 0.001), JPT (Japanese in Tokyo, Japan; *p* = 0.001), KHV (Kinh in Ho Chi Minh City, Vietnam; *p* < 0.001), SAS (South Asian; *p* = 0.014), BEB (Bengali in Bangladesh; *p* = 0.003), GIH (Gujarati Indian in Houston, TX; *p* = 0.007), ITU (Indian Telugu in the UK; *p* = 0.001). We found no statistical significance across the following populations: AMR (American; *p* = 0.826), CLM (Colombian in Medellin, Colombia; *p* = 0.544), MXL (Mexican Ancestry in Los Angeles, California; *p* = 0.883), PEL (Peruvian in Lima, Peru; *p* > 0.999), PUR (Puerto Rican in Puerto Rico; *p* = 0.112), EUR (European; *p* = 0.621), CEU (Utah residents with Northern and Western European ancestry; *p* = 0.642), FIN (Finnish in Finland; *p* = 0.642), GBR (British in England and Scotland; *p* = 0.391), IBS (Iberian populations in Spain; *p* = 0.912), TSI (Toscani in Italy; *p* = 0.242), PJL (Punjabi in Lahore, Pakistan; *p* = 0.079) y STU (Sri Lankan Tamil in the UK; *p* = 0.084).

Throughout the rs622342 genotypic frequency comparative analysis, results were consistent respect to other samples, except for PJL (Punjabi in Lahore, Pakistan; *p* = 0.039) and STU (Sri Lankan Tamil in the UK; *p* = 0.024); Supplementary Material S2.

The comparison of allelic frequencies of SNP rs72552763 against samples from gnomAD exomes r2.1.1 (Harrison et al., 2024), revealed significant statistical differences for AFR (African/African American; *p* < 0.001), ASJ (Ashkenazi Jewish; *p* < 0.001), EAS (East Asian; *p* < 0.001), FIN (Finnish; *p* < 0.001), NFE (Non-Finnish European; *p* = 0.001), OTH (Other; *p* < 0.001), and SAS (South Asian; *p* < 0.001), whereas our sample’s allelic frequencies were no different from AMR (Latino; *p* = 0.073).

In the NCBI ALFA database we found differences respect to EUR (European; *p* = 0.002), AFR-O (African Others; *p* < 0.001), EAS (East Asian; *p* < 0.001), AFR-AMR (African American; *p* < 0.001), LAT1 (Latin American 1; *p* < 0.001), OAS (Other Asian; *p* < 0.001), SAS (South Asian; *p* < 0.001), AFR (African; *p* < 0.001), AS (Asian; *p* < 0.001), OTH (Other; *p* < 0.001), while there were no differences respect to LAT2 (Latin American 2; *p* = 0.370), Supplementary Material S3.

### 3.2 Inference analysis t0 vs. t1 by genotypes

The inferential analysis of the variables compared a set of 3 groups (GAT/GAT, GAT/del, and del/del in rs72552763) and another set of 2 groups for the dominant genotypic model (GAT/GAT vs. GAT/del + del/del) between t0 and t1 (Table 4). There was a significant difference in HbA1c levels among the 3 genotypes of rs72552763 by time 0 (*p* = 0.007). HbA1c levels of these 3 genotypes by t0 and t1 reported statistical significance GAT/GAT (*p* < 0.001), GAT/del (*p* < 0.001), and del/del (*p* = 0.048). The dominant genotypic model group reported statistical significance in HbA1c levels at time 0 (GAT/GAT vs. GAT/del + del/del; *p* = 0.002). Δt1-t0 for GAT/GAT was 0.50 (−0.50, 1.25; *p* < 0.001) and 0.50 (0.02, 1.40) for GAT/del + del/del (*p* < 0.001). Statistical significance was found by metformin dosage (mg/kg/day) among GAT/del patients when comparing t1 and t0 (*p* = 0.001). The dominant genotypic model assessment revealed statistical significance by final metformin dosage (mg/kg/day) between GAT/GAT vs. GAT/del + del/del (*p* = 0.041) and also by Δt1-t0 metformin dose (mg/kg/day) where GAT/GAT reported a change of 0.38 (−0.46, 7.20) and del (GAT/del + del/del) reported 6.87 (0.55, 13.10), (*p* = 0.018) (Table 4). The inferential analysis of the variables compared a set of 3 groups (A/A, A/C, and C/C) in rs622342 and another set of 2 groups for the dominant genotypic model (A/A, A/C + C/C) between t0 and t1 (Table 5). There was a significant difference in HbA1c levels among the 3 genotypes of rs622342 by time 0 (*p* = 0.017). HbA1c levels of these 3 genotypes by t0 and t1 reported statistical significance A/A (*p* < 0.010), A/C (*p* < 0.001), and C/C (*p* = 0.004). The dominant genotypic model group reported non statistical significance in HbA1c levels at time 0 (A/A vs. A/C + C/C); (*p* = 0.13). Δt1-t0 for A/A was 0.040 (−0.10–0.08; *p* = 0.010) and 0.90 (0.07, 1.40) for A/C + C/C; (*p* < 0.001) (Table 5). Statistical significance was found by metformin dosage (mg/kg/day) among A/C patients when comparing t1 and t0 (*p* < 0.001). The dominant genotypic model assessment revealed statistical significance by Δt1-t0 final metformin dosage (mg/kg/day) in A/C + C/C (*p* < 0.001) where reported a change of 4.24 (0.23, 12.60) and A/A reported 0.13 (−0.64, 7.74), *p* = 0.346 (Table 5).

**TABLE 4:** Clinical variable inference T0 vs T1, grouped by genotype and dominant genotype model of rs72552763.

		Genotype analysis					Dominant genotype model			
	Genotype	Time 0	Time 1	Δ_t1-t0_	*p*-value	Genotype	Time 0	Time 1	Δ_t1-t0_	*p*-value
**HbA1c, %**	**GAT/GAT**	6.10 (5.70 – 6.30)	6.80 (6.00 – 7.45)	0.50 (−0.50, 1.25)	<0.001*	GAT/GAT	6.10 (5.70 – 6.30)	6.80 (6.00 – 7.45)	0.50 (−0.50, 1.25)	<0.001*
	**GAT/del**	6.40 (6.20 – 6.60)	7.30 (6.40 - 8.00)	1.0 (0.10, 1.40)	<0.001*	GAT/del + del/del	6.40 (6.20 – 6.60)	7.15 (6.50 – 8.00)	0.50 (0.02, 1.40)	<0.001*
	**del/del**	6.50 (6.20 – 6.70)	7.0 (6.70 – 7.20)	0.50 (0.00, 0.50)	0.048*					
	***p*-value**	0.007*	**0.171**	0.617			0.002*	0.072	0.680	
**Fasting glucose, mg/dl**	**GAT/GAT**	122.00 (109.50 – 150.50)	120.00 (101.00 – 135.00)	−11.00 (−42.50, 8.50)	0.062	GAT/GAT	122.00 (109.50 – 150.50)	120.00 (101.00 – 135.00)	−11.00 (−42.50, 8.50)	0.062
	**GAT/del**	132.00 (109.50 – 143.50)	122.50 (103.50 – 149.00)	−9.50 (−25.75, 27.75)	0.453	GAT/del + del/del	132.00 (112.50 – 150.00)	128.50 (111.50 – 149.00)	−10.00 (−25.75, 18.00)	0.351
	**del/del**	133.00 (126.75 – 161.75)	134.00 (126.00 – 143.50)	−10.50 (−23.75, 14.00)	0.546					
	***p*-value**	0.668	0.195	0.585			0.820	0.170	0.450	
**Metformin dose, mg/kg/day**	**GAT/GAT**	19.60 (12.10 – 23.50)	20.90 (12.20 – 28.80)	0.38 (−0.46, 7.20)	0.202	GAT/GAT	19.60 (12.10 – 23.50)	20.90 (12.20 – 28.80)	0.38 (−0.46, 7.20)	0.202
	**GAT/del**	13.90 (10.80 – 22.30)	26.50 (21.30 – 30.90)	9.95 (0.55, 12.80)	0.001*	GAT/del + del/del	16.90 (11.00 – 23.20)	27.20 (21.30 – 32.30)	6.87 (0.55, 13.10)	<0.001*
	**del/del**	23.10 (17.60 – 26.70)	31.60 (22.30 – 38.70)	2.57 (−0.36, 14.90)	0.250					
	***p*-value**	0.197	0.064	0.057			0.660	0.041*	0.018*	

Median and interquartile ranges (p25-p75). Δ final-initial: t1-t0, Difference between time 1 and time 0; *Statistical significance (p < 0.05).

**TABLE 5:** Clinical variable inference T0 vs T1, grouped by genotype and dominant genotypic model of rs622342.

		Genotype analysis					Dominant genotype model			
	Genotype	Time 0	Time 1	Δ_t1-t0_	*p*-value	Genotype	Time 0	Time 1	Δ_t1-t0_	*p*-value
**HbA1c, %**	A/A	6.10 (5.80 – 6.40)	6.50 (6.00 – 7.10)	0.40 (−0.10, 0.80)	0.010*	A/A	6.10 (5.80 – 6.40)	6.50 (6.00 – 7.10)	0.40 (−0.10, 0.80)	0.010*
	A/C	6.20 (5.90 – 6.50)	7.20 (6.10 – 8.00)	1.10 (0.10, 1.40)	<0.001*	A/C + C/C	6.25 (6.17 – 6.60)	7.20 (6.50 – 8.00)	0.90 (0.07, 1.40)	<0.001*
	C/C^a^	6.50 (6.20 – 6.70)	7.20 (6.70 – 7.90)	0.50 (0.20, 1.45)	0.004*					
	*p*-value	0.017*	0.071	0.278			0.130	0.027*	0.120	
Fasting glucose, mg/dl	A/A	123.00 (111.00 – 152.00)	117.00 (102.00 – 136.00)	−11.00 (-55.00, 7.00)	0.073	A/A	123.00 (111.00 – 152.00)	117.00 (102.00 – 136.00)	−11.00 (-55.00, 7.00)	0.073
	A/C	130.00 (107.25 – 145.25)	125.00 (99.50 – 138.50)	−15.00 (-30.00, 10.50)	0.263	A/C + C/C	132.00 (111.00 – 149.00)	127.00 (109.25 – 141.50)	−10.00 (-25.25, 14.00)	0.307
	C/C	132.00 (123.00 – 156.00)	130.00 (122.00 – 154.00)	−5.00 (-23.00, 20.00)	0.916					
	*p*-value	0.899	0.264	0.553			0.810	0.320	0.340	
**Metformin dose, mg/kg/day**	A/A	20.00 (11.90 – 23.80)	22.30 (12.20 – 28.70)	0.13 (−0.64, 7.74)	0.346	A/A	20.00 (11.90 – 23.80)	22.30 (12.20 – 28.70)	0.13 (−0.64, 7.74)	0.346
	A/C	13.90 (10.80 – 20.10)	24.00 (14.70 – 29.90)	5.04 (0.23, 12.00)	0.001*	A/C + C/C	17.00 (11.00 – 22.90)	26.40 (17.20 – 31.50)	4.24 (0.23, 12.60)	<0.001*
	C/C	22.00 (16.60 – 28.70)	29.90 (22.30 – 34.20)	2.57 (−0.73, 14.90)	0.129					
	*p*-value	0.117	0.143	0.196			0.540	0.280	0.072	

Median and interquartile ranges (p25-p75). Δ final-initial: t1-t0, Difference between time 1 and time 0; *Statistical significance (p < 0.05).

### 3.3 Survival analysis

**FIGURE 1:**
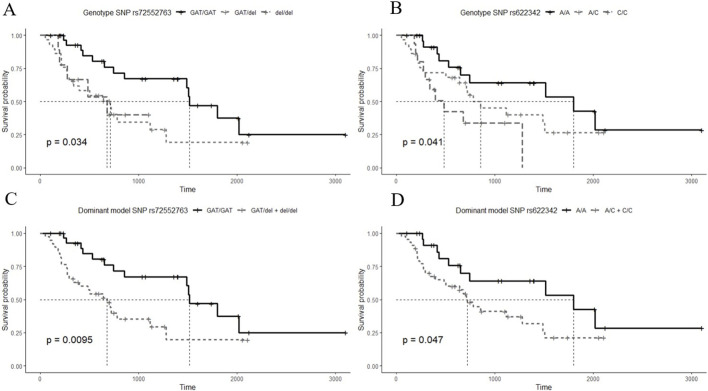
Survival time Kaplan-Meier curves by HbA1c (≥7%) non-control according to studied SNPs. **(A)** curves for the 3 genotypes of rs72552763 [GAT/GAT = 1,513.00 (95% CI.: 1,198.90–1827.09), GAT/del = 709.00 (95% CI.: 360.25–1,057.74), del/del = 677.00 (95% CI.: 160.783–1,193.21); *p* = 0.034], **(B)** curves for the 3 genotypes of rs622342 [A/A = 1798.00 (95% CI.: 410.58–3,185.41), A/C = 854.00 (95% CI.: 323.01–1,384.98), C/C = 484.00 (95% CI.: 251.30–716.69); *p* = 0.041]. **(C)** curves for dominant genotypic model of rs72552763 [GAT/del + del/del = 677.00 (95% CI.: 360.25–1,057.74); *p* = 0.0095]. **(D)** curves for dominant genotypic model of rs622342 [A/C + C/C = 718 (95% CI.: 545.51–890.48); *p* = 0.047]. The thinnest dotted line represents the survival median. *p*-value corresponds to the Log-Rank test.

Kaplan-Meier curves for the 3 genotypes of rs72552763 are shown in Figure 1. Median survival until non-control status in terms of days was 1,513.00 (95% CI.: 1,198.90–1827.09) for GAT/GAT, 709.00 (95% CI.: 360.25–1,057.74) for GAT/del, and 677.00 (95% CI.: 160.783–1,193.21) for del/del, *p* = 0.034 (Panel A). Patients were also grouped by dominant genotypic models whose Kaplan-Meier curves are shown in Figure 1, where the median survival until non-control status was 677.00 (95% CI.: 360.25–1,057.74) for del (GAT/del + del/del), *p* = 0.009 (Panel C). The analogue median survival across rs622342 genotypes was 1798.00 (95% CI.: 410.58–3,185.41) for A/A, 854.00 (95% CI.: 323.01–1,384.98) for A/C, and 484.00 (95% CI.: 251.30–716.69) for C/C, *p* = 0.041 (Panel B). Curves for the dominant genotypic model in rs622342 [AC + CC = 718 (95% CI.: 545.51–890.48); *p* = 0.047] (Panel D). A multivariate analysis of Cox proportional hazards model revealed statistical significance amongst GAT/GAT (*p* = 0.034) throughout the whole sample (n = 69). Other SNPs of *SLC22A1*, *SLC22A2,* and *SLC22A3* were analysed too, but the results are not displayed because we found no statistical significance. We have added a figure for curves by genotypes of rs316019 in *SLC22A2* and rs2076828 in *SLC22A3* as Supplementary Image S1 and another one on *SLC22A1* SNPs (rs2282143, rs594709, rs628031, and rs6833369) as Supplementary Image S2. We found no statistical significance therein either. The Cox univariate analysis revealed statistical significance amongst GAT/GAT [HR = 0.407 (95% CI.: 0.202–0.818); *p* = 0.011] and final metformin dose [HR = 1.040 (95% CI.: 1.003–1.079); *p* = 0.034]. The same univariate model revealed an association between final metformin dose [HR = 1.061 (95% CI.: 1.003–1.123); *p* = 0.039] and del (GAT/del + del/del). The multivariate analysis throughout the whole sample (n = 69) revealed a statistically significant presence of GAT/GAT in rs72552763 [0.430 (95% CI.: 0.197–0.939); *p* = 0.034] (Table 6). The multiple model performed on rs622342 yielded similar results [0.392 (95% CI.: 0.169–0.910); *p* = 0.029] by A/A. Statistical significances were found only for final metformin dosage (mg/kg/day) in the global sample model and the A/C + CC models, both univariate [HR = 1.078 (95% C.I.: 1.028–1.131); *p* = 0.001] and multivariate [HR = 1.094 (95% CI.: 1.030–1.161); *p* = 0.003] (Table 7).

**TABLE 6:** Univariate and multivariate Cox proportional hazards models for non-control of HbA1c for SNP rs72552763.

	Univariate		Multivariate	
	H.R. (95% C.I.)	*p*-value	H.R. (95% C.I.)	*p*-value
Total sample (n = 69)				
GAT/GAT genotype	0.407 (0.202–0.818)	0.011*	0.430 (0.197–0.939)	0.034*
Initial metformin dose, mg/kg/day	1.022 (0.981–1.064)	0.285	1.008 (0.959–1.061)	0.731
Initial weight, kg	0.992 (0.972–1.014)	0.510	1.004 (0.982–1.027)	0.666
Final metformin dose, mg/kg/day	1.040 (1.003–1.079)	0.034*	1.034 (0.990–1.079)	0.127
GAT/GAT carriers (n = 31)				
Initial metformin dose, mg/kg/day	1.034 (0.965–1.108)	0.335	1.051 (0.964–1.146)	0.256
Initial weight, kg	1.002 (0.973–1.033)	0.872	1.006 (0.976–1.038)	0.671
Final metformin dose, mg/kg/day	1.005 (0.949–1.065)	0.851	0.982 (0.913–1.057)	0.632
GAT/del + del/del carriers (n = 38)				
Initial metformin dose, mg/kg/day	1.023 (0.972–1.078)	0.374	1.011 (0.948–1.079)	0.722
Initial weight, kg	0.984 (0.957–1.013)	0.285	1.009 (0.974–1.045)	0.602
Final metformin dose, mg/kg/day	1.061 (1.003–1.123)	0.039*	1.070 (0.999–1.146)	0.053

H.R., hazard ratio; 95% C.I., 95% Confidence interval; * Statistical significance (*p* < 0.05).

**TABLE 7:** Univariate and multivariate Cox proportional hazards models for non-control of HbA1c for SNP rs622342.

	Univariate		Multivariate	
	H.R. (95% C.I.)	*p*-value	H.R. (95% C.I.)	*p*-value
Total sample (n = 69)				
A/A genotype	0.482 (0.231–1.007)	0.052	0.392 (0.169–0.910)	0.029*
Initial metformin dose, mg/kg/day	1.022 (0.981–1.064)	0.285	1.009 (0.958–1.062)	0.733
Initial weight, kg	0.992 (0.972–1.014)	0.510	1.001 (0.980–1.022)	0.898
Final metformin dose, mg/kg/day	1.040 (1.003–1.079)	0.034*	1.043 (1.000–1.089)	0.045*
A/A carriers (n = 25)				
Initial metformin dose, mg/kg/day	0.999 (0.927–1.077)	0.991	1.025 (0.935–1.124)	0.591
Initial weight, kg	0.973 (0.921–1.028)	0.331	0.977 (0.922–1.036)	0.442
Final metformin dose, mg/kg/day	0.985 (0.918–1.057)	0.682	0.975 (0.892–1.066)	0.581
A/C + C/C carriers (n = 44)				
Initial metformin dose, mg/kg/day	1.044 (0.992–1.100)	0.096	1.021 (0.961–1.086)	0.492
Initial weight, kg	0.990 (0.969–1.013)	0.417	1.014 (0.990–1.038)	0.234
Final metformin dose, mg/kg/day	1.078 (1.028–1.131)	0.001*	1.094 (1.030–1.161)	0.003*

H.R., Hazard ratio; 95% C.I., 95% Confidence interval; * Statistical significance (*p* < 0.05).

## 4 Discussion

### 4.1 Survival analysis

We think the most relevant result of our study are the Kaplan-Meier curves for the 3 genotypes of rs72552763 shown in Figure 1. Median survival until non-control status in terms of days was 1,513.00 for GAT/GAT, 709 for GAT/del, and 677 for del/del, *p* = 0.034 (Panel A). Patients were also grouped by dominant genotypic models whose Kaplan-Meier curves are shown in Figure 1, where the median survival until non-control status was 677 for del (GAT/del + del/del), *p* = 0.009 (Panel C). The analogue median survival across rs622342 genotypes was 1,798.00 for A/A, 854.00 for A/C, and 484.00 for C/C, *p* = 0.041 (Panel B). Curves for the dominant genotypic model in rs622342 [AC + CC = 718; *p* = 0.047] (Panel D). A multivariate analysis of Cox proportional hazards model revealed statistical significance amongst GAT/GAT (*p* = 0.034) throughout the whole sample (n = 69). As far as we know, this is the first survival analysis of HbA1c using this design, which employs univariate and multivariate models accounting for these two *SLC22A1* SNPs and exclusively focusing on DMT2 patients undergoing metformin monotherapy. Such design could be applied to other populations to corroborate or rebuke our results, and also to unveil particular therapeutic response traits based on genetic differences across the world.

### 4.2 Allelic and genotypic frequencies

Considering Mexico’s multiethnicity (Jimenez-Sanchez, 2003), it is important to conduct supplemental comparative analyses using genetic reports from such databases as 1,000 Genomes, gnomAD exomes r2.1.1, and NCBI ALFA (Harrison et al., 2024). That is why we compared allelic and genotypic frequencies of rs622342 and rs72552763. It should be noted that the information gathered on these polymorphisms’ frequencies does not belong to a DMT2 population, which might have skewed the selection. The frequencies differ greatly between our sample and several other world populations Supplementary Materials S2, S3. Apparently, this is the first inference of its kind on rs622342 and rs72552763. Allelic and genotypic frequencies within our sample were no different from AMR (America) and EUR (Europe) groups reported in the 1,000 Genomes Project Phase 3. Based upon these results we can confirm that the frequency of ancestral allele A in rs622342 is not preserved among DMT2 Mexican-Mestizo individuals. This ancestral allele is most prevalent (84.1%) among AFR (African), more specifically, in MSL (Mende in Sierra Leone). Our comparison of rs72552763 allelic frequencies revealed our sample was different from other populations, except for AMR (American) from gnomAD exomes r2.1.1 and LAT2 (Latin American 2) from NCBI ALFA. The del allele was most frequent in AMR (21.7%) and LAT2 (27.2%). Following these two groups, del was reported by NFE (Non-Finnish European, 13.8%) and EUR (European, 14.9%), Supplementary Material S3. According to IDF 2021, nine of the 10 most affected countries in terms of DMT2 prevalence by region and ethnicity for every 100,000 inhabitants are in the Americas. 1.- Brazil, 2.- Canada (First Nation People), 3.- Mexico (Unknown), 4.- US (Black), 5.- US (American Indian), 6.- US (Navajo Nation People), 7.- US (Hispanics), 8.- US (Whole population), 9.- US (Asian/Pacific Islander), and 10.- Kuwait. Genetic predisposition, societal-economic disparity, and health service access may well contribute to these differences (International Diabetes Federation, 2021).

### 4.3 Discrepancies respect to previous studies

To our knowledge, Zhou et al. (2009) is the only work where a survival analysis and a Cox proportional hazards test have been used to study metformin’s therapeutic failure through rs72552763 genotype grouping. Findings on rs72552763 in *SLC22A1* by Zhou indicate this variant has no influence on HbA1c control over time. Our results do not match Zhou et al. (2009), where no statistical differences were found between Kaplan-Meier curves across rs72552763 genotypes grouped by dominant genotypic model. Conversely, our analyses yielded different Kaplan-Meier curves for rs72552763 and rs622342 genotypes, and the dominant genotypic model for rs72552763 (GAT/GAT vs. GAT/del + del/del) and rs622342 (A/A vs. A/C + CC) as well. We believe this discrepancy may be due to case status. Our considered case criterion was the first monitoring moment (t0), where the clinical record contained evidence of laboratory studies indicating the patient achieved HbA1c levels equal or higher than 7% (t1); whereas the status for Zhou et al. (2009), is metformin’s therapeutic failure, either the point in time when a patient requires a metformin dosage of 2g/day or an additional therapy. In the face of this discrepancy, another possible explanation is the studied population. Zhou et al. (2009), studied European patients from Scotland, whilst we studied Mestizo patients from North America, more specifically, Mexico. Unmeasured variables such as ethnic diversity, nutrition habits, and exercising may have an important effect on diabetes control (International Diabetes Federation, 2021). This argument is grounded on DMT2 prevalence differences between populations of America and Europe. Moreover, we did not study the ancestral component, which may influence complex traits and disease incidence (Sohail et al., 2023). Since the observed effects could be attributed to a disregarded variant, our study’s limitations manifest the necessity to further study other variants involved in metformin pharmacokinetics and HbA1c control across different populations. A survey by Tarasova et al. (2012), In opposition to results by Ortega-Ayala et al. (2022) and Menjivar et al. (2020), assessed the possible association of *SLC22A1, SLC22A2* and *SLC47A1* polymorphisms with metformin’s adverse effects on 246 DMT2 patients from the Genome Database of Latvian Population. They found no differences in HbA1c levels when comparing genotypes in rs72552763 through additive (*p* = 0.66), dominant (*p* = 0.48), and recessive (*p* = 0.50) models. While there were no statistically significant results, the lowest HbA1c levels were observed by GAT/GAT (GAT/GAT: 7.80 ± 3.27, GAT/del:8.56 ± 2.02 and del/del: 8.43 ± 2.08) (Tarasova et al., 2012). These results do not match our own, which may be due to the different study designs and aims: Tarasova et al. (2012), carried out a cross-sectional study including cases and controls according to metformin adverse reactions, whilst our study is longitudinal and accounted for HbA1c non-control. Although the discrepancy is clear and Tarasova et al. (2012), found no statistically significant differences in HbA1c levels across rs72552763 genotypes, the lowest HbA1c levels were observed by GAT/GAT carriers. In 2015, Mahrooz et al. (2015), studied the effect of rs72552763 on metformin therapeutic response within a sample of 108 DMT2 Iranian patients undergoing metformin monotherapy. They performed a dominant genotypic model which found no metformin response association with rs72552763 (*p* = 0.069), although GAT/GAT was prevalently responsive (Mahrooz et al., 2015). They came to the conclusion that rs72552763 may impact fasting glucose levels as opposed to HbA1c, furthermore, the effect of this SNP might not transpire over time. Our results do not match these since we report GAT/GAT to be associated with a longer control period [HR = 0.407 (95% CI.: 0.202–0.818); *p* = 0.011]. Nevertheless, the association analysis in Mahrooz et al., responder (n = 48) vs. non-responder (n = 59), revealed no statistical significance and the highest response prevalence was found by GAT/GAT (66.7%). This statistical significance absence may be due the sample’s size, where a larger group could yield more conclusive results, as acknowledged by the authors themselves.

### 4.4 Coincidences with other studies

In a sample of 159 Danish patients from the South Danish Diabetes Study, Christensen et al. (2011), found that del in rs72552763 was statistically associated with a decrease in metformin’s steady-state. Steady-state is a pharmacological parameter which regulates drug administration frequency, thereby, according to Christensen et al. (2011), this aforementioned decrease may entail the need of a greater dose to achieve a therapeutic effect. Our results suggest that, when comparing metformin dose (mg/kg/day) at t0 vs. t1, del in rs72552763 (*p* < 0.001) and C in rs622342 (*p* < 0.001), receive the largest dosage, which is consistent with Christensen et al. (2011). In 2015, Umamaheswaran et al. (2015), assessed the impact of rs622342 in *SLC22A1* over metformin’s clinical efficacy in a sample of 122 DMT2 patients undergoing metformin monotherapy in Southern India. They found that C in rs622342 was more likely to be metformin unresponsive, where the response parameter was a decrease of HbA1c ≥ 0.5% 12 weeks after monitoring started, thereby concluding that rs622342 seems to significantly affect metformin response among DMT2 patients from Southern India (Umamaheswaran et al., 2015). These results are consistent with our own, where C in rs622342 appears associated with HbA1c non-control among DMT2 Mexican patients over time.

Another study on 265 DMT2 Mexican-Mestizo by Menjivar et al. (2020), assessed the impact on oral hypoglycemiant response. Through a diplotype analysis they found an association between glycaemic control and GAT in rs72552763 and A in rs622342 among patients undergoing metformin monotherapy [OR = 3.06 (95% C.I.: 1.161–8.100); *p* = 0.026] (Menjivar et al., 2020). Their study concluded there is an interaction between rs72552763 and rs622342 in *SLC22A1* and metformin therapeutic response among Mexican-Mestizo DMT2 patients (Menjivar et al., 2020). These results are consistent with our own, where alleles del in rs72552763 and C in rs622342, both belonging to *SLC22A1*, report an HbA1c non-control risk over time. A previous study by our research team (Ortega-Ayala et al., 2024), used a machine learning model which raises the possibility of predicting HbA1c levels in DMT2 patients undergoing metformin monotherapy based on dosage (mg/kg/day) and genotype. On this larger sample we found that the greatest increase in metformin dose was reported by del in rs72552763 and only C in rs622342 reported a significant mg/kg/day dose (*p* < 0.001), while A/A reported no significant increase (*p* = 0.346).

We performed univariate and multivariate proportional hazards models on the grouped sample (rs72552763 and rs622342), where subgrouping was set according to dominant genotypic models GAT/GAT vs. GAT/del + del/del (Table 6) and A/A vs. A/C + C/C (Table 7). The Cox univariate analysis revealed an association between HbA1c non-control and final metformin dose (mg/kg/day) as opposed to an association between GAT/GAT and control, however, only the genotype sustained a significant association in the multivariate analysis (Table 6). These results suggest that GAT/GAT is statistically associated with metformin monotherapy response over time (*p* = 0.034), wich means that a patient carrying this genotype will reach non-control status later (HbA1c ≥ 7.0%) than patients carrying del.

These results concur with transversal surveys such as Ortega-Ayala et al. (2022) and Menjivar et al. (2020), and also Mahrooz et al. (2015), since they suggest that over time the largest proportion of patients under glycaemic control (HbA1c <7.0%) will carry GAT/GAT in rs72552763. The subgrouped dominant genotypic model of rs72552763 revealed no variable associated with non-control by GAT/GAT, but there was an association between metformin final dose (mg/kg/day) and therapeutic failure over time by del in rs72552763 (*p* = 0.039) (Table 6). These results suggest that del carriers are prescribed a greater metformin dose (mg/kg/day) to achieve HbA1c control. This concurs with Christensen et al.; 2011, because patients carrying del in rs72552763 may present a lower steady-state which implies larger doses to achieve therapeutic effect. The Cox model revealed similar cases by rs622342, which was therein associated with therapeutic response. Our data suggest that A/A in rs622342 will take longer to reach non-control when compared to C (*p* = 0.029) (Table 7). The subgroup analysis of A/C + C/C revealed an association with metformin dose (mg/kg/day), suggesting that C carriers require heavier dosages to achieve therapeutic effect. This coincides specifically with Menjivar et al., 2020, who conducted a diplotype analysis of rs72552763 and rs622342 for GAT and A respectively, where these patients were found the most responsive to metformin monotherapy (Menjivar et al., 2020).

The results of the present study, together with previously reported evidence across the world, suggest that rs72552763 and rs622342 can be used as biomarkers of therapeutic response in metformin monotherapy for recently diagnosed DMT2 patients. We can also suggests that carriers of the minor allele del in both rs72552763 and rs622342 will require a larger metformin dose to achieve glycaemic control, thus reducing DMT2 implicated risks over time. Our results also suggest the possibility of a reduced-function transporter by del in rs72552763 as compared to GAT/GAT. This is supported by Sundelin et al. (2017), who reported a hepatic distribution volume (Vd) 33.9% lower by del/del as opposed to GAT/GAT and 21.42% lower when compared to GAT/del, when measuring ^11^C-metformin uptake through positron emission tomography (PET)-tracer. This indicates that the hepatic distribution volume will diminish as the rs72552763 deletion appears, although, based on our Figure 1A, we cannot dismiss that del (GAT/del + del/del) might equally impact transporting functions in homozygous and heterozygous carriers of this polymorphism. Metformin carries out its main action mechanism over the hepatocyte, by diminishing hepatic glucose production (Staiger et al., 2015), therefore a lower transport unto this site would entail a reduced clinical effect among patients carrying del. Moreover, metformin plasmatic concentrations could be higher as opposed to GAT/GAT, which increases adverse reaction risks by del, as reported by Dujic et al. (2016). Another OCT1-transported drug is morphine (Balyan et al., 2017). Studying a sample of paediatric postoperative tonsillectomy patients anaesthetised with morphine, Balyan et al. (2017), found that individuals carrying del/del were associated with morphine-induced postoperative respiratory depression. This report buttresses the hypothesis that del in rs72552763 diminishes OCT function. Again, it might be possible to find higher morphine plasmatic concentrations among del/del carriers, which would increase adverse reaction risks such as opioid-induced respiratory depression. Becker et al. (2011), reported that carriers of the minor allele C in rs622342 were prescribed a higher dose of levodopa, an anti-Parkinson drug also transported by OCT1. In contrast with rs72552763 where a 3 bp deletion occurs, in rs622342 an intronic region base change takes place, which might entail reduced-function transporter, *SLC22A1* expression changes, or linkage imbalance respect to a functional SNP. According to Figure 1B, the mechanism could be a decreased expression of *SLC22A1*, since the presence of minor allele C involves a shorter HbA1c non-control timelapse.

### 4.5 Strengths and limitations

The main strengths of our study are its methodological design and the employed statistical analysis techniques. The sampling enabled random participation; the longitudinal design enabled a survival analysis from the moment the patient reached non-control status; Cox proportional hazards tests were applied to rs72552763 and rs622342 of *SLC22A1* over the whole sample and also in dominant genotypic model data subsets, including both univariate and multivariate models. The most relevant limitation of our work is the sample’s size, whose small dimensions would not allow similar inferences vis-à-vis *SLC22A2* and *SLC22A3.* Furthermore, this study is based upon a Mexican-Mestizo population, which carries a wide genetic variability (Sohail et al., 2023). Pharmacogenetics is relevant for drug prescription pursuing personalised therapy (Estévez-Paredes et al., 2024). While there is still discrepancy between pharmacogenetic biomarkers and their use in clinical practice (Estévez-Paredes et al., 2024), data contributed by this study show a possible implementation of metformin response pharmacogenetic biomarkers for DMT2 Mexican-Mestizo patients, a further step towards personalised medicine. However, more observational studies and clinical surveys including multivariate analyses and machine learning should be conducted to reduce discrepancies between the biomarker’s presence and its clinical response, thus enhancing its reliability within public health systems.

## Data Availability

The data presented in this study are available on request via the corresponding author. These data are not publicly available because the patients and researchers are bound to an agreement establishing that only the head of the study and Mexican health authorities shall have access to them, in accordance with the presidential decree of 16 April 2015, sanctioning the General Law on Transparency and Access to Public Information.
