# Supplementary figures and images for: Longitudinal assessment of SNPs rs72552763 and rs622342 in SLC22A1 over HbA1c control among Mexican-Mestizo diabetic type 2 patients
Source: Front Pharmacol. 2024 Sep 30;15:1433519. doi: 10.3389/fphar.2024.1433519 (PMC11471661; doi:10.3389/fphar.2024.1433519)

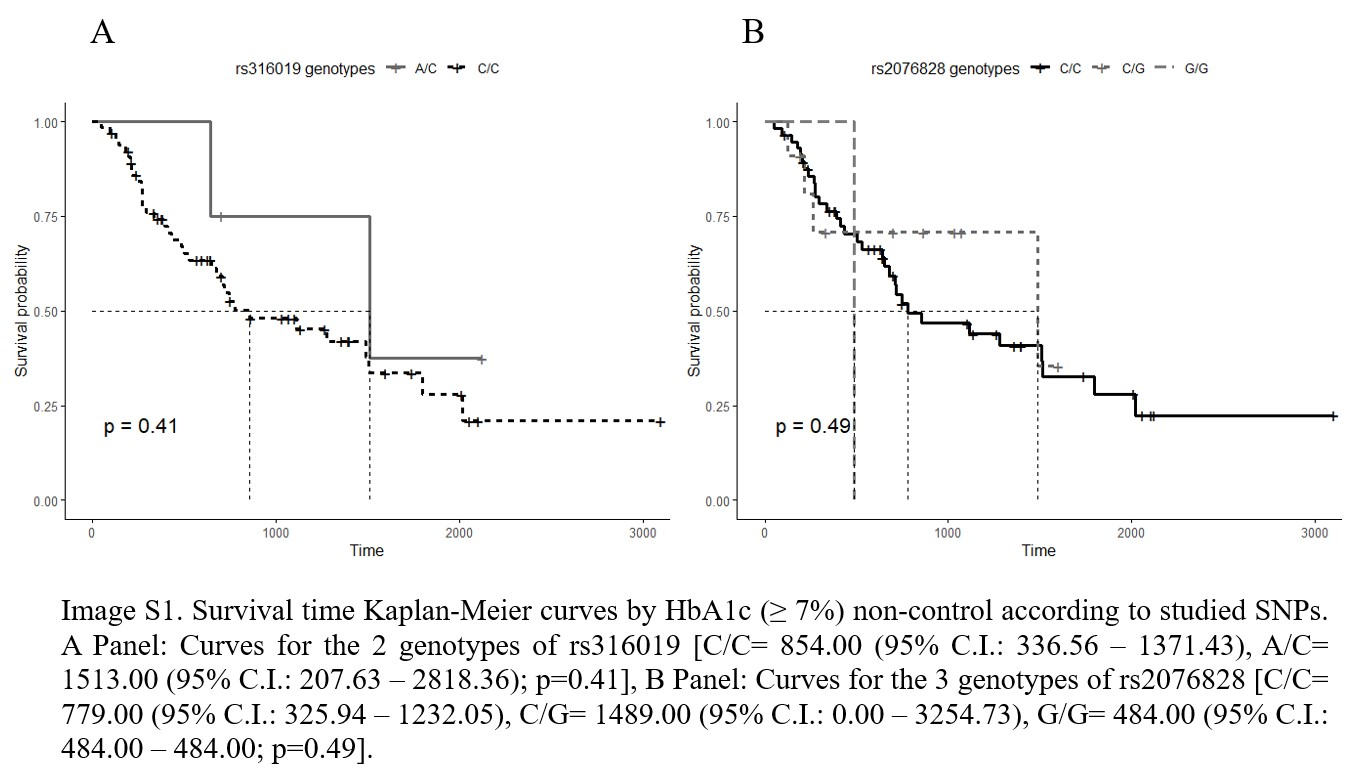

Supplement: Supplementary file 1 [file Image1.tiff]

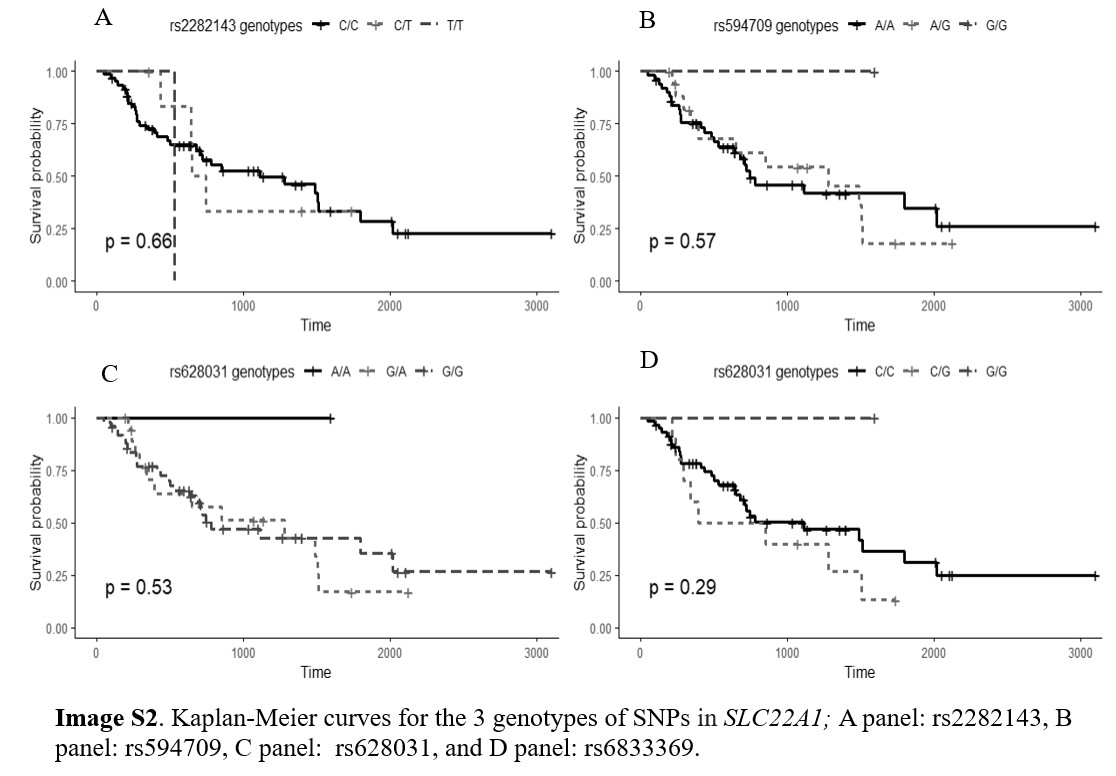

Supplement: Supplementary file 5 [file Image2.tiff]
