# Supplementary material for: Longitudinal assessment of SNPs rs72552763 and rs622342 in SLC22A1 over HbA1c control among Mexican-Mestizo diabetic type 2 patients
Source: Front Pharmacol. 2024 Sep 30;15:1433519. doi: 10.3389/fphar.2024.1433519 (PMC11471661; doi:10.3389/fphar.2024.1433519)
Supplement: Supplementary file 3 [file Table2.pdf]

S2. Compared allelic and genotypic frequencies of rs622342 between DMT2 Mexican-Mestizo patients and other world populations (1000 Genomes database).

	Allele: Count (frequency)			Genotype: Count (frequency)			
Population	A	C	P[ǂ]	AA	AC	CC	P[§]
**Our study**	**79 (0.572)**	**59 (0.427)**	**-**	**25 (0.362)**	**29 (0.420)**	**15 (0.217)**	-
**ALL**	3709 (0.741)	1299 (0.259)	**0.019***	1419 (0.567)	871 (0.348)	214 (0.085)	**0.003***
**AFR**	1084 (0.820)	238 (0.180)	**<0.001***	448 (0.678)	188 (0.284)	25 (0.038)	**<0.001***
ACB	160 (0.833)	32 (0.167)	**<0.001***	66 (0.688)	28 (0.292)	2 (0.021)	**<0.001***
ASW	97 (0.795)	27 (0.205)	**0.001***	37 (0.607)	23 (0.377)	1 (0.016)	**<0.001***
ESN	166 (0.838)	32 (0.162)	**<0.001***	70 (0.707)	26 (0.263)	3 (0.030)	**<0.001***
GWD	180 (0.796)	46 (0.204)	**<0.001***	74 (0.655)	32 (0.283)	7 (0.062)	**<0.001***
LWK	158 (0.798)	40 (0.202)	**0.001***	63 (0.636)	32 (0.323)	4 (0.040)	**<0.001***
MSL	143 (0.841)	27 (0.159)	**<0.001***	62 (0.729)	19 (0.224)	4 (0.047)	**<0.001***
YRI	180 (0.833)	36 (0.167)	**<0.001***	76 (0.704)	28 (0.259)	4 (0.037)	**<0.001***
**AMR**	416 (0.599)	278 (0.401)	0.826	138 (0.398)	140 (0.403)	69 (0.199)	0.862
CLM	98 (0.521)	90 (0.479)	0.544	27 (0.287)	44 (0.468)	23 (0.245)	0.522
MXL	76 (0.594)	52 (0.406)	0.883	26 (0.406)	24 (0.375)	14 (0.219)	0.784
PEL	98 (0.576)	72 (0.424)	>0.999	30 (0.353)	38 (0.447)	17 (0.200)	0.946
PUR	144 (0.692)	64 (0.308)	0.112	55 (0.529)	34 (0.327)	15 (0.144)	0.064
**EAS**	855 (0.848)	153 (0.152)	**<0.001***	365 (0.724)	125 (0.248)	14 (0.028)	**<0.001***
CDX	161 (0.866)	25 (0.134)	**<0.001***	70 (0.753)	21 (0.226)	2 (0.022)	**<0.001***
CHB	179 (0.869)	27 (0.131)	**<0.001***	80 (0.777)	19 (0.184)	4 (0.039)	**<0.001***
CHS	178 (0.848)	32 (0.152)	**<0.001***	74 (0.705)	30 (0.286)	1 (0.010)	**<0.001***
JPT	165 (0.793)	43 (0.207)	**0.001***	66 (0.635)	33 (0.317)	5 (0.048)	**<0.001***
KHV	172 (0.869)	26 (0.131)	**<0.001***	75 (0.758)	22 (0.222)	2 (0.020)	**<0.001***
**EUR**	622 (0.618)	384 (0.382)	0.621	191 (0.380)	240 (0.477)	72 (0.143)	0.399
CEU	122 (0.616)	76 (0.384)	0.642	39 (0.394)	44 (0.444)	16 (0.162)	0.628
FIN	122 (0.616)	76 (0.384)	0.642	37 (0.374)	48 (0.485)	14 (0.141)	0.353
GBR	117 (0.643)	65 (0.357)	0.391	37 (0.407)	43 (0.473)	11 (0.121)	0.178
IBS	119 (0.556)	95 (0.444)	0.912	29 (0.271)	61 (0.570)	17 (0.159)	0.104
TSI	142 (0.664)	72 (0.336)	0.242	49 (0.458)	44 (0.411)	14 (0.131)	0.212
**SAS**	732 (0.748)	246 (0.252)	**0.014***	277 (0.566)	178 (0.364)	34 (0.070)	**0.001***
BEB	134 (0.779)	38 (0.221)	**0.003***	51 (0.593)	32 (0.372)	3 (0.035)	**<0.001***
GIH	157 (0.762)	49 (0.238)	**0.007***	59 (0.573)	39 (0.379)	5 (0.049)	**<0.001***
ITU	163 (0.799)	41 (0.201)	**0.001***	67 (0.657)	29 (0.284)	6 (0.059)	**<0.001***
PJL	135 (0.703)	57 (0.297)	0.079	50 (0.521)	35 (0.365)	11 (0.115)	**0.039***
STU	143 (0.701)	61 (0.299)	0.084	50 (0.490)	43 (0.422)	9 (0.088)	**0.024***

[ǂ] Pearson's Chi-squared test with Yates' continuity correction.
[§]Test de Chi cuadrado de Pearson basado en simulación de Monte Carlo con 2000 replicaciones.
1000 genomes Project population: AFR (African), ACB (African Caribbean in Barbados), ASW (African Ancestry in Southwest US), ESN (Esan in Nigeria), GWD (Gambian in Western Division, The Gambia), LWK (Luhya in Webuye, Kenya), MSL (Mende in Sierra Leone), YRI (Yoruba in Ibadan, Nigeria), AMR (American), CLM (Colombian in Medellin, Colombia), MXL (Mexican Ancestry in Los Angeles, California), PEL (Peruvian in Lima, Peru), PUR (Puerto Rican in Puerto Rico), EAS (East Asian), CDX (Chinese Dai in Xishuangbanna, China), CHB (Han Chinese in Beijing, China), CHS (Southern Han Chinese, China), JPT (Japanese in Tokyo, Japan), KHV (Kinh in Ho Chi Minh City, Vietnam), EUR (European), CEU (Utah residents with Northern and Western European ancestry), FIN (Finnish in Finland), GBR (British in England and Scotland), IBS (Iberian populations in Spain), TSI (Toscani in Italy), SAS (South Asian), BEB (Bengali in Bangladesh), GIH (Gujarati Indian in Houston, TX), ITU (Indian Telugu in

the UK), PJL (Punjabi in Lahore, Pakistan), STU (Sri Lankan Tamil in the UK).
