# Supplementary material for: Longitudinal assessment of SNPs rs72552763 and rs622342 in SLC22A1 over HbA1c control among Mexican-Mestizo diabetic type 2 patients
Source: Front Pharmacol. 2024 Sep 30;15:1433519. doi: 10.3389/fphar.2024.1433519 (PMC11471661; doi:10.3389/fphar.2024.1433519)
Supplement: Supplementary file 4 [file Table3.pdf]

S3. Compared allelic and genotypic frequencies of rs72552763 between DMT2 Mexican-Mestizo patients and other world populations (gnomAD exomes r2.1.1 y NCBI ALFA).

Population	Allele: Count (frequency)		
	GAT	del	P[ǂ]
**Our study**	**91 (0.659)**	**47 (0.340)**	-
gnomAD exomes r2.1.1			
ALL	130814 (0.879)	18010 (0.121)	**<0.001***
AFR	11042 (0.954)	530 (0.046)	**<0.001***
AMR	11349 (0.783)	3141 (0.217)	0.073
ASJ	3417 (0.912)	331 (0.088)	**<0.001***
EAS	11500 (0.999)	8 (0.001)	**<0.001***
FIN	13673 (0.890)	1697 (0.110)	**<0.001***
NFE	64828 (0.862)	10404 (0.138)	**0.001***
OTH	2753 (0.886)	355 (0.114)	**<0.001***
SAS	12252 (0.888)	1544 (0.112)	**<0.001***
NCBI ALFA			
ALL	22264 (0.866)	3450 (0.134)	**0.001***
EUR	16155 (0.851)	2833 (0.149)	**0.002***
AFR-O	115 (0.958)	5 (0.042)	**<0.001***
EAS	99 (0.990)	1 (0.010)	**<0.001***
AFR-AMR	3096 (0.940)	198 (0.060)	**<0.001***
LAT1	132 (0.904)	14 (0.096)	**<0.001***
LAT2	444 (0.728)	166 (0.272)	0.370
OAS	44 (1.000)	0 (0.000)	**<0.001***
SAS	88 (0.898)	10 (0.102)	**<0.001***
AFR	3211 (0.941)	203 (0.059)	**<0.001***
AS	143 (0.993)	1 (0.007)	**<0.001***
OTH	2091 (0.904)	223 (0.096)	**<0.001***

[ǂ] Pearson's Chi-squared test with Yates' continuity correction.

gnomAD exomes r2.1.1 population: AFR (African/African American), AMR (Latino), ASJ (Ashkenazi Jewish), EAS (East Asian), FIN (Finnish), NFE (Non-Finnish European), OTH (Other), SAS (South Asian).

NCBI ALFA population: EUR (European), AFR-O (African Others), EAS (East Asian), AFR-AMR (African American), LAT (Latin American 1), LAT2 (Latin American 2), OAS (Other Asian), SAS (South Asian), AFR (African), AS (Asian), OTH (Other).
