# Supplementary material for: Longitudinal assessment of SNPs rs72552763 and rs622342 in SLC22A1 over HbA1c control among Mexican-Mestizo diabetic type 2 patients
Source: Front Pharmacol. 2024 Sep 30;15:1433519. doi: 10.3389/fphar.2024.1433519 (PMC11471661; doi:10.3389/fphar.2024.1433519)
Supplement: Supplementary file 2 [file Table1.pdf]

S1. Genotypic and allelic frequencies and HWE of SNPs in *SLC22A1, SLC22A2* and *SLC22A3.*

Gene/protein	SNP	Genotype	Genotypic frequency n (%)	Expected frequency n (%)	Allele frequency n (%)	HWE χ^2 *p*-value	HWE $\chi^{2\parallel}$ *p*-value	HWE *exact test*[ǂ] *p*-value
SLC22A1/ OCT-1	rs12208357	C/C	66 (95.65%)	66.03(95.70%)	C: 135 (97.83%)<br>T: 3 (2.17%)	0.034<br>0.850	6.766<br>>0.999	0.00<br>>0.999
		C/T	3 (4.35%)	2.93 (4.25%)				
		T/T	0 (0.00%)	0.03 (0.05%)				
	rs2282143	C/C	61 (88.41%)	60.29 (93.48%)	C: 129 (93.48%)<br>T: 9 (6.52%)	1.946<br>0.162	0.245<br>0.244	0.757<br>0.241
		C/T	7 (10.14%)	8.41 (12.19%)				
		T/T	1 (1.44%)	0.29 (0.43%)				
	rs594709	A/A	50 (72.46%)	50.45 (73.11%)	A: 118 (85.51%)<br>G: 20 (14.49%)	0.190<br>0.662	0.011<br>>0.999	0.00<br>>0.999
		A/G	18 (26.08%)	17.10 (24.78%)				
		G/G	1 (1.44%)	1.45 (2.10%)				
	rs628031	G/G	49 (71.01%)	49.60 (71.88%)	G: 117 (84.78%)<br>A: 21 (15.22%)	0.311<br>0.576	0.033<br>0.695	0.00<br>>0.999
		G/A	19 (27.53%)	17.80 (25.80%)				
		A/A	1 (1.44%)	1.60 (2.32%)				
	rs6833369	C/C	58 (84.05%)	57.52 (83.36%)	C: 126 (91.30%)<br>G: 12 (8.70%)	0.525<br>0.468	0.019<br>>0.999	0.593<br>0.411
		C/G	10 (14.49%)	10.96 (15.88%)				
		G/G	1 (1.44%)	0.52 (0.76%)				
SLC22A2/ OCT-2	rs306019	C/C	65 (94.20%)	65.05 (94.29%)	C: 134 (97.10%)<br>A: 4 (2.89%)	0.061<br>0.804	3.411<br>>0.999	0.00<br>>0.999
		C/A	4 (5.79%)	3.88 (5.63%)				
		A/A	0 (0.00%)	0.05 (0.08%)				
SLC22A3/ OCT-3	rs2076828	C/C	57 (82.60%)	56.61 (82.05%)	A: 125 (90.58%)<br>C: 13 (9.42%)	0.299<br>0.584	0.027<br>>0.999	0.536<br>0.462
		C/G	11 (15.94%)	11.78 (17.07%)				
		G/G	1 (1.44%)	0.61 (0.89%)				

SLC, Solute carrier; SNP, Single Nucleotide Polymorphism; HWE, Hardy-Weinberg Equilibrium, χ^2, Xi statistic.
[‖] Chi-square statistic with continuity correction with 1000 permutations, [ǂ] statistic exact test with 1000 permutations.
